# Minoxidil and bilateral central serous chorioretinopathy in an adolescent girl: relationship or causality?

**DOI:** 10.22336/rjo.2025.22

**Published:** 2025

**Authors:** Juan Manuel Correa Alvarez, Sara Turizo Mejia, Mauricio Arango Hurtado

**Affiliations:** Department of Ophthalmology, CES University, CLOFAN Clinic, Medellin, Colombia

**Keywords:** central serous chorioretinopathy, minoxidil, case report, CSC = central serous chorioretinopathy, SRF = Subretinal fluid, VA = visual acuity, BCVA = best-corrected visual acuity, OCT = Optica Coherence Tomography, RPE = retinal pigment epithelium

## Abstract

**Objective:**

This report describes a case of bilateral central serous chorioretinopathy linked to cosmetic minoxidil use in an adolescent girl, highlighting clinical findings, progression, and implications for practice.

Materials and methods: Case report.

**Results:**

A 14-year-old girl with no significant medical history reported several weeks of blurred vision in both eyes. She had been using 2% topical minoxidil on her eyebrows and eyelashes without a prescription for the past 6 months. The initial examination revealed that the best-corrected visual acuity (BCVA) was 20/80 in the right eye and 20/25 in the left eye, with no abnormalities noted in the anterior segment. Fundus evaluation and OCT confirmed bilateral serous retinal detachment. Given the patient’s unsupervised minoxidil use, a causal link to central serous chorioretinopathy (CSC) was suspected. After discontinuing the drug, the subretinal fluid (SRF) resolved, and visual acuity fully recovered within three months, supporting the association between minoxidil and choroidal neovascularization (CNV), not CSC.

**Discussion:**

Topical minoxidil has been widely used due to its vasodilatory, anti-inflammatory, anti-androgenic, and trichogenic properties, primarily through its induction of the Wnt/β-catenin signaling pathway. Despite its benefits, adverse effects such as hypotension, pruritus, and hypertrichosis are common, and isolated reports have linked its use to CSC, with symptoms typically resolving after discontinuation of the drug in most case studies. Proposed mechanisms for this association include systemic absorption leading to increased choroidal vascular permeability and disruption of retinal homeostasis. To our knowledge, this is the first reported case in the literature of bilateral CSC linked to cosmetic minoxidil use in an adolescent girl, with complete resorption of SRF and recovery of visual acuity following discontinuation of the drug.

**Conclusion:**

CSC linked to topical minoxidil use is rare and requires early detection for prompt discontinuation and recovery. Further research is needed to confirm this link, understand the underlying mechanisms, and develop more effective treatments.

## Introduction

Central serous chorioretinopathy (CSC) is a pathology within the spectrum of pachychoroid disease, characterized by the accumulation of subretinal fluid (SRF) due to increased choroidal permeability that promotes the passage of fluid through the retinal pigment epithelium (RPE) [1]. It is considered the fourth most common non-surgical retinopathy and usually affects men between 20 and 60 years of age, while its presentation in adolescents is anecdotal. Minoxidil has been recognized as an effective treatment for androgenetic alopecia for over two decades, with local adverse reactions to its topical application primarily limited to irritant and allergic contact dermatitis. However, a few recent reports have linked the use of topical minoxidil, a medication commonly prescribed for androgenic alopecia, to the development of CSC [2-5]. This report describes a case of bilateral CSC in an adolescent girl following the cosmetic use of 2% minoxidil, highlighting key clinical findings, disease progression, and implications for clinical practice.

## Case report

**Fig. 1:**
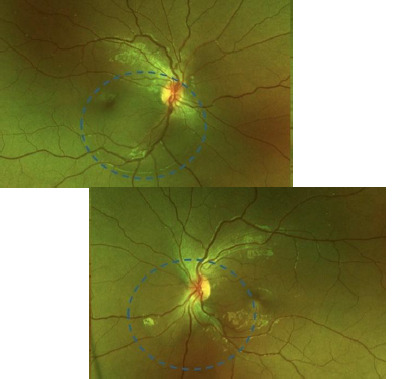
The fundus photographs of both eyes reveal the presence of peripapillary subretinal fluid and fluid in the macular area (blue dotted line)

**Fig. 2:**
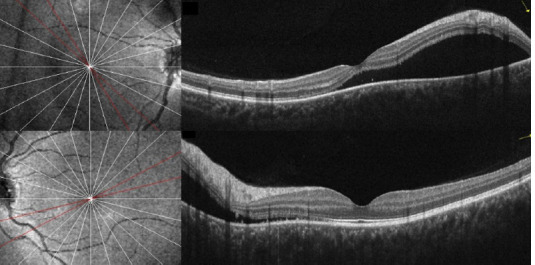
Initial macular OCT scans from both eyes depict SRF with subfoveal involvement in OD (top) and peripapillary involvement in OS (bottom)

**Fig. 3:**
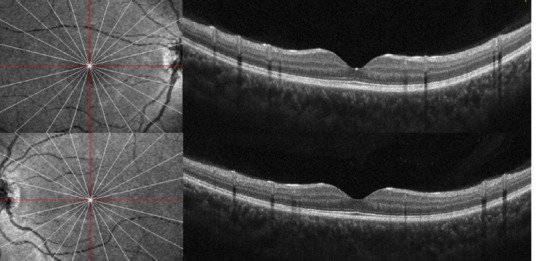
Three-month follow-up macular OCT scans of both eyes showed complete resolution of the subretinal fluid

A 14-year-old female patient with no relevant pathologic history was consulted for several weeks of blurred vision in both eyes. She admitted to the non-prescribed use of topical minoxidil 2% on her eyebrows and eyelashes during the last 6 months. On initial evaluation, the best corrected visual acuity (BCVA) was 20/80 in the right and 20/25 in the left eye. No abnormalities were noted in the anterior segment. Fundus evaluation (**Fig. 1**) revealed a bilateral serous retinal detachment, which was confirmed by optical coherence tomography (OCT) (**Fig. 2**). Following the diagnosis of CSC in the context of unsupervised minoxidil use, a causal link was suspected, and discontinuation of the drug was recommended. Three months later, the SRF was resolved entirely (**Fig. 3**), and visual acuity had fully recovered, reinforcing the association between minoxidil and the development of the condition.

## Discussion

Topical minoxidil, available in 2% and 5% concentrations, is FDA-approved for androgenic alopecia and acts as a vasodilator with anti-inflammatory and anti-androgenic effects through its induction of the Wnt/β-catenin signaling pathway [6]. While common adverse effects include hypotension, pruritus, skin erythema, facial swelling, and hypertrichosis, there have been anecdotal reports linking minoxidil to CSC [2-5]. In 2012, Scarinci et al. reported the first case of a 37-year-old man with CSC who had been undergoing treatment for androgenetic alopecia with 2% minoxidil solution for eight months. One month after the drug was discontinued, the subretinal fluid completely resolved [2]. Similarly, in 2020, Venkatesh et al. reported a case of a 41-year-old male treated for androgenic alopecia with topical 5% minoxidil, who experienced complete resolution of symptoms and subretinal fluid one month after discontinuing minoxidil and taking oral eplerenone 50 mg/day for four weeks [3]. Mankumar et al. also documented a CSC case related to applying 5% topical minoxidil, successfully managed by discontinuing the drug and performing focal laser photocoagulation [4].

Proposed mechanisms for this association include systemic absorption leading to increased choroidal vascular permeability, direct RPE toxicity, and disruption of Müller cells, which affects retinal homeostasis [4,5]. The authors mentioned earlier have also highlighted these mechanisms as contributing factors to the development of CSC in patients undergoing minoxidil therapy.

The standard first-line treatment for acute CSC is observation and management of risk factors, as most cases resolve naturally with good visual outcomes [1]. In our patient’s case, discontinuing the drug led to complete resorption of the SRF and visual improvement to 20/20 in both eyes, suggesting a potential causal link between minoxidil use and CSC. To our knowledge, this is the first reported case in the literature of bilateral central serous chorioretinopathy linked to cosmetic minoxidil use in an adolescent girl.

## Conclusion

CSC related to topical minoxidil use is an uncommon condition that requires early detection to facilitate the immediate discontinuation of the drug, leading to prompt anatomical and functional improvement. However, further research is needed to validate this association, deepen our understanding of the underlying mechanisms, and develop more effective therapeutic strategies.
